# Assessing Scientific Soundness and Translational Value of Animal Studies on DPP4 Inhibitors for Treating Type 2 Diabetes Mellitus

**DOI:** 10.3390/biology10020155

**Published:** 2021-02-16

**Authors:** Nuno Henrique Franco, Sonia Batista Miranda, Nóra Kovács, Attila Nagy, Bùi Quốc Thiện, Flávio Reis, Orsolya Varga

**Affiliations:** 1 Laboratory Animal Science Group, IBMC—Instituto de Biologia Molecular e Celular, Universidade do Porto, Rua Alfredo Allen 208, 4200-135 Porto, Portugal; nfranco@ibmc.up.pt (N.H.F.); soniabatistamiranda@outlook.com (S.B.M.); 2 Instituto de Investigação e Inovação da Universidade do Porto, Rua Alfredo Allen 208, 4200-135 Porto, Portugal; 3 Department of Public Health and Epidemiology, Faculty of Medicine, University of Debrecen, Kassai út 26, 4028 Debrecen, Hungary; kovacs.nora@med.unideb.hu; 4 Faculty of Public Health, University of Debrecen, Kassai út 26, 4028 Debrecen, Hungary; nagy.attila@sph.unideb.hu; 5 Faculty of Medicine, University of Debrecen, Egyetem Square 1, 4032 Debrecen, Hungary; buiquocthien1996@gmail.com; 6 Institute of Pharmacology and Experimental Therapeutics, and Coimbra Institute for Clinical and Biomedical Research (iCBR), Faculty of Medicine, University of Coimbra, 3000-548 Coimbra, Portugal; freis@fmed.uc.pt; 7 Center for Innovative Biomedicine and Biotechnology (CIBB), University of Coimbra, 3004-504 Coimbra, Portugal; 8 Clinical Academic Center of Coimbra (CACC), 3004-504 Coimbra, Portugal; 9 Office for Research Groups Attached to Universities and Other Institutions, Hungarian Academy of Sciences, 1051 Budapest, Hungary

**Keywords:** animal models, predictive validity, type 2 diabetes mellitus, translational medicine, research bias

## Abstract

**Simple Summary:**

The value of animal models to predict human outcomes has been increasingly challenged due to a low rate of translation between preclinical and clinical trials. However, translational failure has been proposed to be at least partly explained by the poor methodological quality of animal studies. We thus retrospectively assessed the predictive value of animal models in Type-2-diabetes research, comparing the same outcome measure (glycaemia) in response to a currently available class of antidiabetic drugs between published clinical trials and animal studies, and assessed methodological quality of the latter. In our sample, both mice and rats performed similarly to humans in response to dipeptidyl peptidase-4 (DPP4) inhibitors. These results, while on animal models of just one disease treated with one drug class, suggest current criticism of animal models may not be entirely warranted, though we found a margin for improvement in the research quality of animal studies.

**Abstract:**

Although there is a wide range of animal models of type 2 diabetes mellitus (T2DM) used in research; we have limited evidence on their translation value. This paper provides a) a comparison of preclinical animal and clinical results on the effect of five dipeptidyl peptidase-4 (DPP4) inhibitors by comparing the pharmaceutical caused glucose changes, and b) an evaluation of methodological and reporting standards in T2DM preclinical animal studies. DPP4 inhibitors play an important role in the clinical management of T2DM: if metformin alone is not sufficient enough to control the blood sugar levels, DPP4 inhibitors are often used as second-line therapy; additionally, DPP-4 inhibitors are also used in triple therapies with metformin and sodium-glucose co-transporter-2 (SGLT-2) inhibitors or with metformin and insulin. In our analysis of 124 preclinical studies and 47 clinical trials, (1) we found no evidence of species differences in glucose change response to DPP4 inhibitors, which may suggest that, for this drug class, studies in mice and rats may be equally predictive of how well a drug will work in humans; and (2) there is good reporting of group size, sex, age, euthanasia method and self-reported compliance with animal welfare regulations in animal studies but poor reporting of justification of group size, along with a strong bias towards the use of male animals and young animals. Instead of the common non-transparent model selection, we call for a reflective and evidenced-based assessment of predictive validity of the animal models currently available.

## 1. Introduction

Globally 422 million adults suffer from diabetes, 90% of them having diabetes mellitus type 2 (T2DM). Given its prevalence and public health impact, T2DM is intensively researched in both academia and industry, an endeavour for which the use of non-human animal (henceforth ‘animals’) models of diabetes has been central [1,2,3,4,5] for giving insight into the underlying pathophysiological mechanisms of the disease, as well as for the development and testing of therapeutic drugs [1,5,6]. Hence, choosing the most appropriate model is of the utmost importance to improve the translatability of results to humans, avoid ignoring potentially valuable treatments, and reduce the risk of basing the entering a test compound into clinical phases on unreliable safety or efficacy data. Furthermore, choosing animal models with greater predictive validity is central, in a time when animal research is under intense scrutiny, not only because of animal welfare concerns but also due to reported low translatability of animal studies [7,8].

There is a wide range of animal models of T2DM available [3,5,9,10,11], which include both spontaneous and induced models. In spontaneous models, T2DM occurs spontaneously in animals, similarly to what is observed in human patients. In regard to induced models, methods include chemical induction (such as administration of streptozotocin or alloxan, with β-cell toxicity features), high-calorie diets, surgery (e.g., by partial pancreatectomy), genetic modification, or a combination of them [1,12]. The informative value of these animal models for human disease may vary, depending on their construct and predictive validity [13]. The construct validity of a model refers to how closely it recapitulates the disease aetiology and mechanisms known in humans, whereas predictive validity expresses how well a model can predict treatment response in humans. Predictive validity can therefore only be verified *a posteriori*, by retrospectively assessing which models had responded to drug treatments in a similar way to human patients. However, discussions of the advantages and caveats of currently available animal models are mostly focused on construct validity (e.g., [2,14]) or more practical issues (e.g., [6]), whereas little information is available on predictive validity on which model selection can be based. We therefore aimed at retrospectively assessing which animal models of T2DM had performed more closely to outcomes in clinical trials, as regards response to a commercially available drug. This follows a previous study comparing fasting glycaemia and HbA1c outcomes between humans and animals in response to rosiglitazone [15]. The validity of animal research also largely depends on methodological standards, and given the reportedly poor methodological quality and low level of detail of published preclinical research [16,17,18], we also assessed adherence to basic measures for minimizing risk of bias—namely sample size justification, blind analysis of outcomes, randomisation, and existence of a conflict of interests’ statement [19,20,21].

There is a wide range of drugs available for dealing with T2DM, most of which being orally administered [22,23]. For the present study, we selected dipeptidyl-peptidase-4 (DPP4) inhibitors (also known as gliptins) as case studies, and of the twelve gliptins available worldwide, we selected the five currently approved by the US Food and Drug Administration or the European Medicines Agency: sitagliptin, saxagliptin, linagliptin, vildagliptin, and alogliptin. DPP4 inhibitors have widely replaced sulfonylureas as second-line therapy after metformin failure or unacceptable side-effects, and many metformin/DPP4 inhibitor fixed-dose combinations are on the market. DPP4 inhibitors are also recommended in the guidelines for triple therapies with metformin and sodium-glucose co-transporter-2 (SGLT-2) inhibitors or with metformin and insulin in later stages of the disease [24]. This class of oral antidiabetic drugs is associated with a low risk of hypoglycemia and has a good overall safety and tolerability profile, being nasopharyngitis and skin lesions the main possible adverse effects, together with the risk of pancreatitis, which has been reported as low [24]. In addition, the VERIFY (Vildagliptin Efficacy in combination with metfoRmIn For earlY treatment of type 2 diabetes) trial showed an advantage of initial metformin-vildagliptin combination therapy when compared to vildagliptin added sequentially to metformin [25]. Although these results have not been proved for other oral drugs and gliptins other than vildagliptin, they reinforce the clinical interest of DPP4 inhibitors, which are already useful as second- and third-line combined therapy.

Our scientific goals were twofold (each divided by sub-tasks):

1. Compare preclinical and clinical outcomes of administration of DPP4 inhibitors.

1.a. Identify which species performed most similarly to humans in response to DPP4 inhibitors treatment, regarding standard diabetes diagnosis parameters, namely fasting glucose and HbA1c levels.

1.b. Compare the predictive value of different strains within each drug class.

1.c. Assess the possible impact of diabetes induction method as a covariate on the predictive capacity of animal models.

2. Evaluate methodological and reporting standards in T2DM preclinical studies.

2.a. Evaluate the likelihood of publication bias.

2.b. Determine the level of detail reported on animal models and methods.

2.c. Assess adherence to basic measures for minimizing the risk of bias.

2.d. Map compliance with animal welfare regulations and reported refinement of animal use.

## 2. Materials and Methods

### 2.1. Search Strategy

Studies published in English in peer-reviewed journals before September 2017 were searched on the MEDLINE, Web of Science, and Embase databases. Human studies were retrieved from MEDLINE, Embase and https://clinicaltrials.gov/ (accessed on 30 September 2017). Search terms are presented in Supplementary file 1 (A). Two reviewers performed the first-stage screening of titles and abstracts based on the research question and its study design, sample, intervention, and outcomes. We selected all studies on animals or humans on the treatment effect of any of the five selected DPP4, reporting fasting glucose levels and/or HbA1c as outcome measures. All studies that did not use T2D models, that had no control group, that did not use animals or humans, that did not report the selected outcome measures, or that were not tested in monotherapy, were excluded.

### 2.2. Data Collection

All references found from the database search were downloaded in the RIS (Research Information System)format and saved on an Endnote^®^ library, and the corresponding full-texts were retrieved. Data were extracted from reading the full papers and entered into a shared, online, purpose-made database. Data extracted on study design included time, route and dose of the drug administration; species and strain of the animal; age and sex of subjects; diet and diabetes induction method. Data on outcomes (glycaemia and HbA1c levels were collected for each treatment group reported, including the number of measurements, and mean. Whenever data were only reported graphically, a digital online ruler was used. The following of avoidance of bias measures was assessed by checking: random assignment to treatment groups; allocation concealment; blinded outcome assessment; “conflict of interests” statement; funding disclosure; unreported ‘missing’ animals (checking whether group sizes varied along the article), and sample size justification (options: power calculation; scientific literature; resource equation; or none). We also collected information on self-reported regulatory compliance, such as reporting of institutional guidelines, legislation and regulations, other guidelines, and project evaluation. Information on animal welfare retrieved included reporting of any refinement measures, the maximum level of glycaemia animals reached, method of euthanasia, and type of endpoint (classified as spontaneous death, humane endpoints or previously defined scientifically-based time-points).

Two reviewers (S.B.M. and O.V.) extracted all data concomitantly. Any questions arising during data collection were discussed with the other team members.

To reduce the impact of bias from poor quality research in our analysis, papers with no control groups (positive, negative, and placebo) were excluded. We also weighted observations proportionally to their precision (reciprocal of squared standard error of treatment effect) during analysis, and a funnel plot was built with this data to check for publication bias.

### 2.3. Data Analysis

The analysis of the quantitative data was stratified according to (i) species and strains (ii) diabetes induction method, (iii) drug administration route, (iv) sex of animals, and (v) diet during the experiments. Data were analysed with the STATA statistical package, and the significance level was set at *p* < 0.05.

The standardized mean differences (SMD) for glucose changes (before and after treatment) were calculated for each intervention drug and the overall DPP4 drug class. Meta-analyses were carried out using STATA IC version 13.0. Student’s t-tests were then used for the given standardized mean differences (SMD, or Cohen’s d) to compare with the related placebos. For example, alogliptin human SMDs were compared with placebo human SMDs. Student’s t-test differences with 95% confidence intervals (CI) were visualized by bar charts and y error bars.

For outcome measure comparison and most other parameters, each treatment group (*n* = 449) from the selected studies was taken as the experimental unit. For the analysis on bias avoidance measures, methodological standards, and regulatory compliance, each paper was deemed the experimental unit, unless otherwise stated (*n* = 124).

## 3. Results

### 3.1. Comparing Preclinical and Clinical Results on the Effect of DPP4 Inhibitors

We report results from a comparison of glucose changes between preclinical gliptins tests and human clinical trials, with the use of meta-regression models for evaluating the drug-induced glucose changes of different species and strains against humans. The impact of diet and diabetes induction method on glucose was also assessed. The PRISMA (Preferred Reporting items for Systematic Reviews and Meta-Analyses) flowchart for the triage of results and of human trials and animal studies is presented as a Supplementary file 1 (B).

Our search strategy originally identified 628 preclinical tests of DPP4 inhibitors (alogliptin—54, linagliptin—90, sitagliptin—269, saxagliptin—66, and vildagliptin—149). Following a triage process applying the inclusion criteria mentioned in Supplementary file 1 (B), 155 rat and mouse studies were included in the analysis (alogliptin—14, linagliptin—20, saxagliptin—11, sitagliptin—74, and vildagliptin—39), with three of them having more than one interventional drug. Due to the very low number of animal studies reporting HbA1c values (*n* = 13), our analysis focused on the glucose values, exclusively. One non-human primate study with two animals and a rabbit study with six animals were excluded as these would not allow a quantitative analysis. Thus, the comparative analysis includes the 124 papers for the two rodent species. Table 1 shows that sitagliptin and vildagliptin were predominantly studied and that no special distribution of animal models was observed with either drug. The most used strain was the Wistar rat, followed by the C57BL/6 mouse. C57B/KsJ-db/db mice were used to test all the five DPP4 inhibitors. Table 2 shows a growing number of DPP4 animal research articles, with a peak in 2016.

The main drug delivery method chosen was oral administration (115/124), with 38/115 administering drugs in the water or food, and the other 77 studies by orogastric gavage. In three studies, the drugs were delivered by injection, and in another three, the route could not be clearly determined. Daily dose in the studies ranged from 0.0003 mg/kg/day to 76.400 mg/kg/day for alogliptin, 3.0 mg/kg/day to 30 mg/kg/day for linagliptin, 0.1 mg/kg/day to 17.5 mg/kg/day for saxaliptin, 1.8 × 10^−5^ mg/kg/day to 11,000 mg/kg/day for sitagliptin, and 0.3 mg/kg/day to 50 mg/kg/day for vildagliptin.

**Figure 1:**
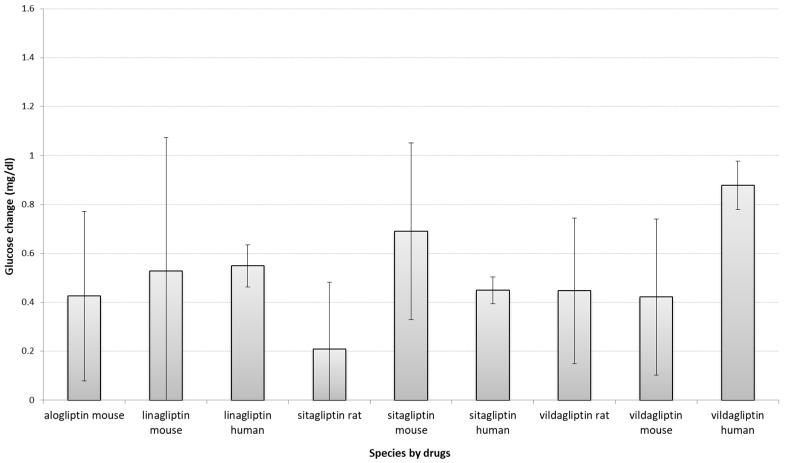
Comparison of absolute glucose changes across species grouped by each DPP4-inhibitors. Bars show the difference between standardized mean differences (SMD, between start-end) of each species stratified by drug and its related placebo (*t*-test between drug SMD and placebo SMD); error bars show the 95% confidence interval (point estimation ±1.96 standard error (SE); SE = standard deviation (SD)/sqrt(n)). The overlap between error bars between species for the same drug shows the lack of significant difference between drug-induced glucose changes. A larger positive glucose change in the figure means a greater reduction in glucose levels. Due to lack of sufficient (at least three studies) studies, the following groups of studies are not shown in the figure: alogliptin rat (*n* = 0) and human (*n* = 1) studies, saxagliptin mouse (*n* = 2), and rat (*n* = 0) studies, linagliptin rat studies (*n* = 2). Data for human saxagliptin studies (*n* = 5) also not presented, as animal studies for comparison were insufficient. Mg/dl—milligrams per decilitre.

In order to identify which models performed most similarly to humans in response to DPP4 inhibitors treatment, we firstly compared before vs after treatment glucose changes across species, grouped by each DPP4-inhibitor drug (Figure 1) by including before-after animal and human studies (*n* = 59 and *n* = 47, retrospectively). Before vs. after studies measure the outcome variable at least at two time points: before the intervention and after the intervention. There were no consistent and significant differences between these changes in human outcomes and either rats and/or mice for alogliptin, linagliptin and sitagliptin. However, the difference between human glucose changes and animals’ for vildagliptin was significant, although not between mice and rats.

**Figure 2:**
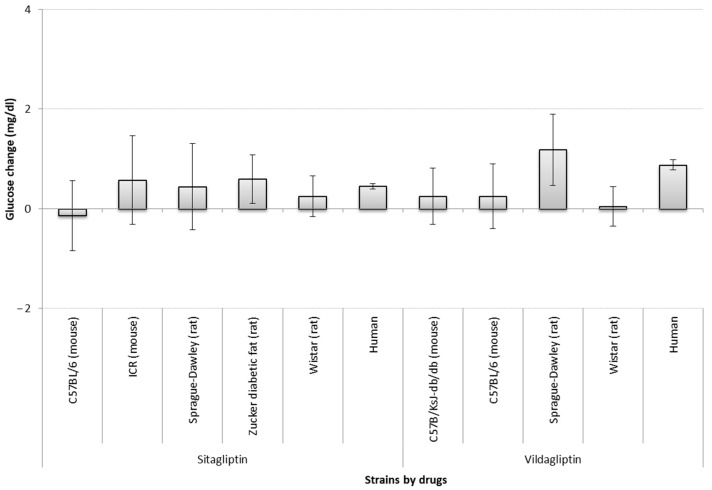
Comparison of absolute glucose changes across strains by sitagliptin and vildagliptin studies. Bars show the difference (*t*-test between drug SMD and placebo SMD) between standardized mean differences (SMD between start-end) of each strain; error bars show the 95% confidence interval (point estimation ±1.96 SE). The overlap between error bars shows the lack of significant difference between glucose changes by strains. A larger positive deviation in the figure means a greater reduction in glucose levels. The list of strains used in models is not complete due to lack of sufficient (at least three studies) information for analysis. Mg/dl—milligrams per decilitre.

Regarding the impact of strains, only for six of the strains (C57BL/6, ICR, Sprague-Dawley, Zucker diabetic fat, Wistar, and C57B/KsJ-db/db) information was sufficient to perform this analysis. Sprague-Dawley rats given vildagliptin, and Zucker diabetic fat models given sitagliptin showed significant glucose reduction after treatment (i.e., a zero change is not included in the confidence interval). However, Wistar models did differ from humans in the vildagliptin group (non-overlapping confidence intervals) (Figure 2).

Regarding diabetes induction method—and including all treatment groups (*n* = 449) from the 124 published animal studies reviewed—diabetic phenotype was achieved most frequently through chemical induction (*n* = 99), followed by high-calorie diet (*n* = 90), a combination of chemical and diet induction (*n* = 53), monogenic background (*n* = 92), polygenic/spontaneous background (*n* = 70), and partial pancreatectomy (*n* = 5). Non-diabetic controls comprised 87 groups, and for 59 of the groups, none of these classifications were applicable.

**Figure 3:**
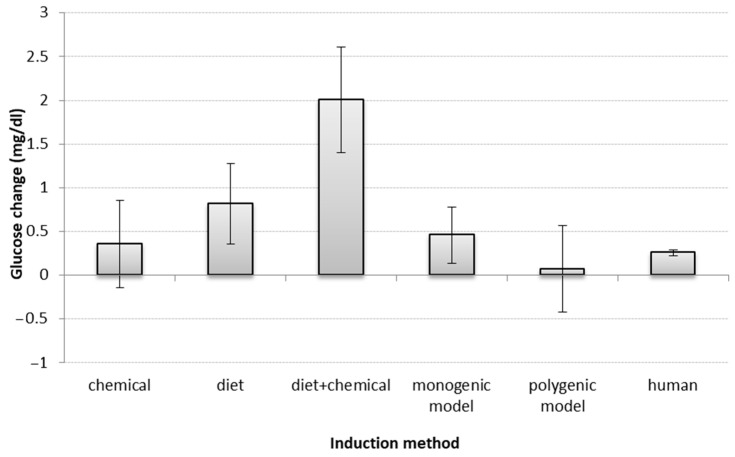
Impact of induction methods on glucose change. Bars show the difference (*t*-test between drug SMD and placebo SMD) between standardized mean differences (SMD between start-end) of each induction method in animals and humans; error bars show the 95% confidence interval (point estimation ±1.96 SE). The overlap between error bars shows the lack of significant difference between glucose changes by induction methods. If the confidence interval includes zero, there is no significant change in glucose level. A larger positive deviation in the figure means a greater reduction in glucose levels. Mg/dl—milligrams per decilitre.

Regarding the impact of induction method on predictive value, 55 articles had sufficient information for the analysis. Similarly to humans, a small glucose reduction was observed for all types of disease induction, overall, though significantly higher in the diet and diet + chemical models (non-overlapping confidence interval (CI)). (Figure 3).

**Figure 4:**
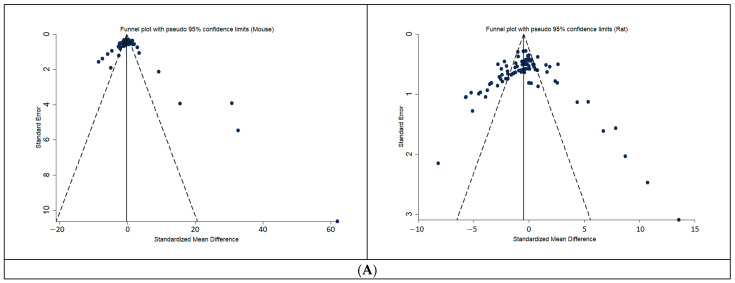

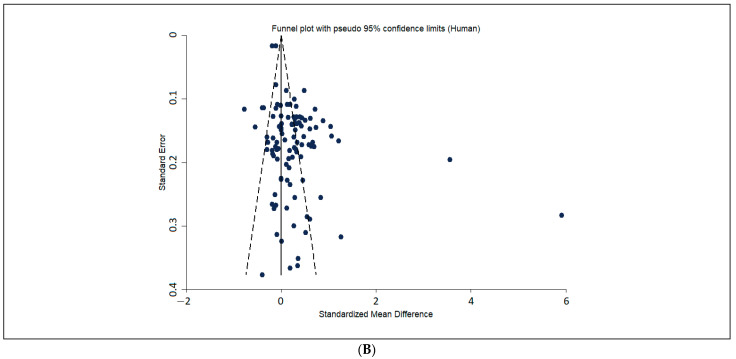
Funnel-plots of the distribution of reported effects in drug treatment groups, for preclinical studies (**A**) humans (**B**). Funnel- plot analysis does not indicate an asymmetry in the distribution of drug-effects of the included animal studies. SE: standard error, SMD: standardized mean differences (positive SMD is good; it means a positive difference between start-end, so smaller end). A positive SMD means a positive difference between the start and end glucose (i.e., smaller end glucose).

The funnel-plot analysis (Figure 4) did not show an asymmetry in the distribution of reported before-after effects in the drug treatment groups of the included animal studies, particularly for the more precise studies.

### 3.2. Methodology and Reporting Standards of the T2DM Preclinical Studies

#### 3.2.1. Level of Detail Reported on Animal Models and Methods

In regard to the level of detail reported, of the 449 treatment groups represented in our sample of 124 papers, information about the diet animals were fed was not retrievable for 40 of these groups. Of the pooled groups for which diet was known, most were either fed a standard diet (52.1%, 213/409) or a fat-rich diet (40.6%, 166/409). (Details on types of diet are shown in Table 3).

**Figure 5:**
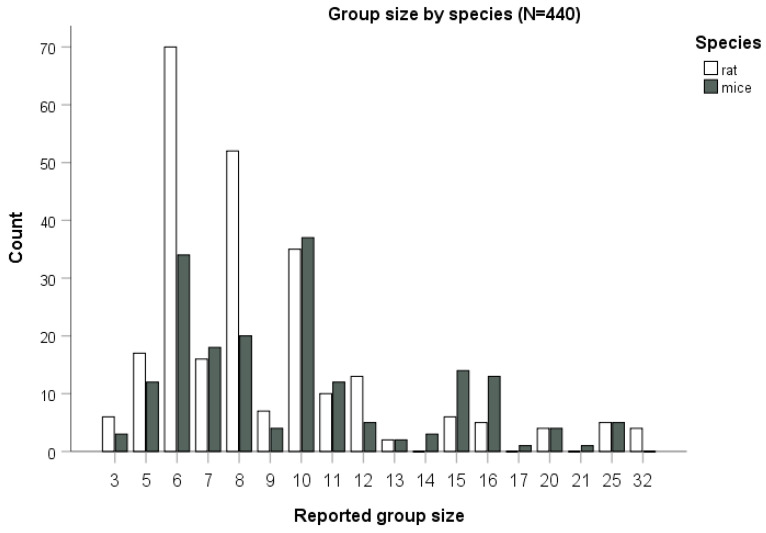
Group size by species. Bars correspond to the number of cases for a total of 440 treatment groups (out of *n* = 449 treatment groups for which this information was available), for rats and mice.

Information on group size was retrievable for 98% of the treatment groups (*n* = 440/449) found in our sample of 124 papers. In papers reporting rat studies (*n* = 69), group size ranged between *n* = 3 and *n* = 32, with an estimated total of 2259 rats in our sample, whereas for mouse studies (*n* = 55) it ranged between *n* = 3 and *n* = 25, with an estimated total of 1886 mice. In one paper, both mice and rats were used (considered here separately). Group size (Figure 5) differed significantly between studies on rats and on mice (Mann-Whitney test, *p* = 0.001). The median group size was eight animals for rats (mean 8.96, SE = 4.81) and of 10 animals for mice (mean 10.0, SD = 4.26), with the group size for both species following a non-normal (Shapiro–Wilk test, *p* < 0.001), skewed (Skewness of 2.68 for rats and 1.26 for mice, Kurtosis of 8.86 for rats and 1.64 for mice) distribution.

The age at the beginning of the experiments was retrievable for all treatment groups in our sample and followed a skewed (with a prevalence of younger animals) non-normal distribution (Shapiro–Wilk test, *p* < 0.001 for both species). The median age for both rat and mouse studies was of 8 weeks (with mean ± SD of 9.6 ± 0.37 for rats and 7.9 ± 0.26 for mice), albeit with significantly different distributions (Mann–Whitney test, *p* < 0.037). Information on the sex of the animals was available for 92.7% of the treatment groups (416/449). Most concerned (86.9%, 390/449) male animals, whereas only 4.9% (22/449) were female groups.

#### 3.2.2. Reported Measures for Minimizing Risk of Bias

Regarding reporting of avoidance of bias measures, 50.8% of the 124 papers reported random allocation of animals to treatment groups. Whenever blinding was reported, it was for histopathological analysis (data not shown), so it would not presumably directly affect the assessment of our outcome of interest (glycaemia). No allocation concealment was reported.

Most (75.8%, 94/124) papers had a statement on funding, eight of which stating that the studies had not been funded. As for self-reported conflict of interests, 28.2% (35/124) of papers did not include any information, while 53.2% (66/124) reported to have no conflict of interests and 18.5% reported a possible conflict of interest (23/124). Only three papers (2.4%) provided a justification for the reported group size, all of which were power calculations. In 15.3% of papers (19/124), group sized varied between what was originally reported in the ‘Methods’ section and what was reported in figures or ‘Results’ section (either reported as lower than originally or as a range, rather than a discrete value), with none of them explicitly mentioning any criteria for the exclusion of animals.

#### 3.2.3. Mapping Self-Reported Compliance with Animal Welfare Regulations and Reported Refinement

The overwhelming majority of papers (92.7%) had some statement on regulatory compliance, namely that the studies had been approved by a third party (national or regional authority, or local ethics committee), followed national legislation, complied with institutional guidelines, or a combination of these. Neither project approval nor stating of compliance with animal welfare regulations were found to significantly improve reporting of refinement measures. Refinement measures were reported in 25.8% of the 124 papers analysed. These comprised measures to hydrate animals (six instances), insulin to prevent severe hyperglycaemia (two instances), and others (24 instances).

The method for euthanasia was not reported in 40.3% of papers. Of the 74 papers reporting it, 66.2% used anaesthetic overdose or another method on anaesthetised animals, whereas decapitation and cervical dislocation on non-anaesthetised animals were used each in 12.2% of known cases, Carbon Dioxide asphyxiation in 8.1%, and in one case animals were reported to be exsanguinated with no other details being provided.

In most (80.6%) studies (109/124), animals were estimated to had been euthanized at predefined time-points, which coincided with the scientific endpoint. In 16 instances, this could not be ascertained, thought death as an endpoint was never explicitly reported to have occurred.

## 4. Discussion

Systematic reviews, meta-analyses and regression studies can help scientists choose the best animal models of disease, by providing evidence of their predictive value in pharmaceutical studies, thus making the evaluation of their translational value more evidence-based. They can also further the 3Rs (for Replacement, Reduction, and Refinement of animal use), as well as allow finding gaps and opportunities to broaden their implementation [26,27]. The predictive value of animal models can be assessed by comparing the same outcome measures (e.g., blood glucose, and HbA1c) between human trials and animal studies. One of the authors of this paper has previously used this approach for diabetes and rosiglitazone [15], where an analysis of 71 animal studies showed variable consistency between animal models with the human reference for glucose and HbA1c treatment effects, and overall a better agreement between treatment effects in rats with the expected values based on human data than in other species. In the present study, we analysed 124 published preclinical studies on mice and rats measuring the effect of five pharmaceuticals of the DPP4 inhibitor class. In summary, we found no significant difference between species in glucose change response to either DPP4 inhibitor, possibly suggesting that studies in mouse and rats models of T2DM are equally useful in predicting drug effects in humans. There was no strong evidence suggesting that induction method or diet type resulted in better prediction, though the effect observed in diet-induced and diet + chemically-induced models was higher than in chemically-induced and spontaneous models. There was good reporting of group size, sex, age, euthanasia method and regulatory compliance on animal care and welfare, but the justification of group size was poorly reported, and there was a very strong bias towards the use of male animals and young animals. The chronic under-reporting of sample size justification is in agreement with the literature [18,28,29] and has worryingly remained unchanged [19]. Indeed, it was deemed an essential item in the revised version of the ARRIVE (Animal Research: Reporting of In Vivo Experiments) guidelines [30], which adoption would likely improve the correct interpretation of results and allow replicability of experiments while facilitating systematic reviews.

In contrast with the regulatory landscape for safety assessment, efficacy testing of pharmaceuticals is scarcely regulated, and there are no strict requirements for pre-clinical testing [31]. Guidance on model selection for efficacy assessment in drug development is nonetheless available in the literature [32,33]. However, very few retrospective assessments of their predictive validity [34] have actually been carried out. Quality of prediction can nonetheless be improved by estimating the relationship between drug efficacy data in humans and animal models quantitatively. Such retrospective assessments [35] and (preferably) prospective human-animal parallel studies may be able to go beyond speculation and provide evidence on predictive value. Based on the present comparison between preclinical and clinical data DPP4 inhibitors, we found no evidence supporting that any rodent species should be preferred, despite—and contrary to most other fields—rats still being the most frequently used species in T2DM research [36]. However, some strains are not able to produce glucose change responses to DPP4 treatment resembling those of humans, including Swiss albino mice. This outbred strain was widely used for several purposes (e.g., toxicology [37] and development [38]), whereas for drug development inbred mice (e.g., C57BL/6) were typically preferred. More data is necessary to understand why outbred rats (e.g., Wistar) resemble human data more closely than outbred mice.

Our data also suggests, and contrary to the common opinion that the best animal model for a disease is one that closely displays its pathophysiology [39], that diabetes induction method apparently does not impose limitations to the translational value of animal models. Despite diet and nutraceutical factors having important effects on diabetes mellitus in humans, the diet-induced rodent models, and contrary to their obvious aetiological relevance, could not provide a higher predictive value [40], and indeed showed a higher effect of drug treatment than found in humans and other animals models. Although the frequently used chemically-induced models seem to have higher predictive value; however, it might be associated with higher animal mortality and other animal welfare problems [41].

The sex and age of the animals were highly reported, in agreement with a systematic review [42] of papers published between 1994–2014 indicating higher reporting of these details for mouse studies on T2DM than in other fields. The high level of reporting in our sample (mostly comprising relatively recent papers), is in agreement with the higher reporting of biological details in recent years [42], as well as with our recent finding that preclinical tests of therapeutic drugs have more details than proof-of-concept studies [16]. Although polygenic models of obesity and T2DM are often considered to be more accurate models of the human condition than monogenic models, the difference between their predictive value could not be confirmed [43].

The sex and age bias found, in agreement with current literature [42], should be cause for concern. If sex differences are non-existent or trivial, selecting only mice of a given sex leads to an ethically unacceptable waste of animals of the other. On the other hand, if these exist and are clinically relevant (which is the case for metabolic diseases, such as T2DM [44]), any sex bias in preclinical research will misrepresent the target population. Furthermore, the predominant choice for young animals contrasts with the evidence that the likelihood of developing T2DM increases after 45 years of age, thus affecting construct validity, and possibly misleading results [45].

The fact that group sizes were relatively small is concerning, considering the typically small effect sizes found in our sample. This is particularly troublesome, since the justification for sample size used is hardly ever reported, either in our sample or elsewhere, with no justifiable reason [16,19]. A power calculation (using G*Power [46]) shows that a *t*-test comparison between treated and untreated animals, with a group size of *n* = 8 animals each, would only identify a true effect as statistically significant (with 80% power and α = 0.05) if the Cohen’s d effect size was higher than 1.4, which is considerably large. For smaller (true) effects, the probability of finding truly-existing treatment differences as being statistically significant (i.e., statistical power) would be lower. Based on data of variability of several outcomes for diabetes and obesity on inbred and outbred mice by Jensen et al. [47] the median group size of *n* = 8 would in most cases only detect (with 80% power and α = 0.05) 1.5 fold differences between two treatment groups. This is even more problematic for the chosen class of drugs, as DPP4 inhibitors have shown a very limited impact in humans as regards reducing blood glucose and HbA1c [48], so expected real effect sizes would be small and only observable with adequate sample sizes. However, this drug class is of clinical interest, and moreover, its potential health benefits go beyond control of glycaemia [49].

Contrary to what has been amply reported for other fields [50] we found no observable publication bias, as a random distribution of reported drug effects on glycaemia variation in drug-treated animals is observable around both sides of the plotted average of the more precise studies, in our funnel plot analysis, for both the two species and humans. The modest effect sizes found may partially be explained by the fact that small—or even no—variations between pre- and post-treatment glycaemic levels within drug-treated groups (the main outcome measure in human trials, against which animal data was compared) can nonetheless signal a drug effect in stabilizing glycaemia, particularly if lower than in untreated groups.

The varying group size found in several papers may be the result of numerous factors: animals being excluded for sound and justifiable reasons (e.g., an injury); lack of resources or time to carry out all tests on all animals; or animals dying from the phenotype or procedures (though the latter was never explicitly mentioned). Regardless of the reason for excluding animals, such reason should be made transparent, so that readers can make an appraisal of the risk of bias. In addition, the uncertainty as regards why animals are excluded has implications for estimating whether any studies allowed animals to die spontaneously from a deleterious phenotype.

The mismatch between the group size stated in the reviewed papers’ ‘Methods’ sections, and the one found throughout the paper—either a lower number or representing it as a range—raises challenges to the interpretation of results, and concern about the reliability of data, especially for small sample sizes. Holman et al. [51] found that random loss of sample size decreased statistical power, whereas biased removal of subjects, including that of outliers, dramatically increased probability of false-positive results. Although there might be a number of justifiable reasons for why animals need to be excluded from an experiment (which should be defined a priori and reported upon publication), the reasons for such variation need to be duly reported, and journal editors and reviewers have the duty to demand this information upon coming across with such mismatch.

The high level of self-reported compliance with animal welfare regulations is in line with previous reports that this has become common practice in recent years [52,53]. However, such reports have been found to have little measurable impact on the implementation of refinement measures [54]. Indeed, and though one could conceivably consider that studies reported to had been appraised and approved by a regulatory body would abide by higher animal welfare higher standards than studies with a self-reported statement of compliance with laws and guidelines, we did not find any significant differences in reporting of refinement measures, or the type of endpoint used.

The preferred method for drug administration was the oral route, which is the closest to the clinical setting. However, in most cases, this was carried out by administering the drug in the food or water, which raises issues concerning the definition of the experimental units, since whenever treatments cannot be independently assigned to each animal but rather administered to the cage as a whole, the cage becomes the experimental unit, which considerably lowers the power of the experiment [55]. However, in none of the papers, analysed was this factored into the analysis.

T2DM models are not typically lethal, so finding that most animals were alive at the end of the experiment was not surprising. However, considering the welfare impact of advanced T2DM in animals, the reported level of implementation of refinement measures is strikingly low. Similar to humans, chronic hyperglycaemia can have a marked impact on animal welfare. Clinical signs of this welfare-impaired state include excessive feeding and drinking (from increased hunger and thirst), as well as polyuria, increased aggressiveness, body weight loss, and reduced activity [56]. Animals experience peripheral neuropathy and allodynia [57], and although it has been proposed that the welfare impact of animals reaching >450 mg/dL glycaemia values raises ethical issues [58], this was observed in 67 of the 449 treatment groups in our sample, often for several weeks (data not shown), which would warrant refinement measures.

During the course of this work, we encountered some limitations. The most relevant was an insufficient number of large sample, high-quality, randomized controlled studies, which could have affected their informative value for this systematic review. Furthermore, the lack of enough non-rodent models prevented any assessment outside the most commonly used rodent species.

Our ability to assess animal welfare standards was also quite limited because of insufficient data, an issue that is transversal to most fields of experimental biomedical research, and should be addressed by the whole scientific community, from authors to journal editors [59,60,61,62].

While we aimed to assess the reporting of the main measures to prevent bias in experimental research (sample size justification, blind analysis of outcomes, randomisation, and having a conflict of interests statement [19,20,21]), we decided to not report information on blinding of observers, since whenever this was reported, it invariably referred to histopathological analysis, whereas our main outcomes were glycaemia levels, objectively measured by an instrument.

## 5. Conclusions

This study proposes an innovative approach for estimating predictive validity in preclinical pharmaceutical studies, by comparing the same outcome responses to the same drugs between different preclinical and clinical studies, and evaluating whether—and if so, to what extent—animal models can predict therapeutic outcomes in humans. As predictive validity has special weight among the validity dimensions, this approach—or other similar approaches with the same objective—should be more common.

Given that public and ethical acceptability of animal research is grounded on a harm-benefit balance, we find it important that retrospective assessment studies also appraise animal welfare and methodological standards, providing a broader picture that includes the care given to the animals and an assessment of the likelihood of benefits (i.e., obtaining credible and replicable results). Regarding methodology, the main concern was the likelihood that studies were underpowered, given the small—and not adequately justified—sample sizes, along with pseudoreplication from treatments being administered cage-wise, and not independently to each animal.

Although collecting data is a relatively straightforward task, access to data is a remarkable bottleneck. For this reason, we stress the need to make background data of preclinical tests readily available, as its analysis will allow animal testing to achieve its full scientific potential and medical relevance. In the future, higher standardization in reporting of preclinical and clinical data may open the way for the automatizing of data collection and analysis, and allow for a more informed, objective and customized animal model selection.

## Figures and Tables

**Table 1:** Distribution of the treatment groups included in the 124 preclinical studies, according to each dipeptidyl peptidase-4 (DPP4) inhibitor and animal species and strain. The total of 210 entries reflects the fact that several papers reported outcomes for more than one of the selected drugs, and/or more than one species or strains. Rats were more represented (*n* = 113) than mice (*n* = 97), in terms of an absolute number of treatment groups included (placebo and untreated groups not included). There was a considerable variety of different models within each species (*n* = 10 for rats and *n* = 19 for mice), limiting the possibility for robust comparative assessment.

Species	Strain	Alogliptin	Linagliptin	Saxagliptin	Sitagliptin	Vildagliptin
**rat**	Fisher344			1	2	
	Goto-Kakizaki (GK)		2	2		2
	Non-diabetic GK		1			
	OLETF **^1^**					2
	Sprague-Dawley		1	1	14	6
	UCD-T2DM **^2^**	2				
	Wistar		1	2	30	30
	Zucker			2		
	Zucker diabetic fat				9	2
	Zucker lean				1	
**mice**	Akita				1	
	apoE−/− **^3^**	3		2		2
	B6129SF1/J				5	
	C/EBPB TG of C57BL/6					2
	C57/DBA.hIAPP				2	
	C57B/KsJ-db/db	5	5	1	2	5
	C57B/KsJ-ob/ob	5			1	3
	C57BL/6	1	6		17	5
	CETP-apoB100 **^4^**				1	
	eNOS knockout C57BL/6 ^**5**^		1			
	fatty liver Shionogi-ob/ob				1	
	ICR ^**6**^	6			5	
	Irs2+/−					1
	Irs2+/+					1
	Irs2−/−					1
	KK-Ay mice	1				1
	LDLR−/− ^**7**^	2				
	NIH/OlaHsd				1	
	swiss albino					2

**^1^** Otsuka Long Evans Tokushima Fatty Rat; **^2^** University of California, Davis Type 2 Diabetes Mellitus Rat; **^3^** Atherosclerosis-prone apolipoprotein E-deficient mouse; **^4^** Cholesteryl Ester Transfer Protein—Apolipoprotein B100 double transgenic mouse; **^5^** Endothelial Nitric Oxide Synthase knockout mouse; ^**6**^ Institute of Cancer Research mouse (outbred); ^**7**^ Low Density Lipoprotein Receptor Knockout Mouse.

**Table 2:** Distribution of our sample, according to the year of publication. This table provides information on the year of publication of the papers included in our sample, showing a gradually growing number of articles published, with a peak in 2016.

Year	2006	2007	2008	2009	2010	2011	2012	2013	2014	2015	2016	2017
Studies on rats	1	0	0	2	2	2	5	13	13	8	15	8
Studies on mice	1	1	1	3	2	8	7	7	7	8	8	3
Total	2	1	1	5	4	10	12	20	20	16	23	11

**Table 3:** Reported diet. The table shows the frequency and proportion of reported diet from all 449 treatment groups (drug and controls) gathered from our sample of 124 papers.

Diet Type	Frequency	Percent
Not reported	40	8.9
Normal chow	213	47.4
High-fat	166	37.0
Low-fat	5	1.1
High fat and high sugar	19	4.2
Other	6	1.3

## Data Availability

The datasets generated during and/or analysed during the current study are available from the corresponding author on reasonable request.

## Supplementary Materials

The following are available online at https://www.mdpi.com/2079-7737/10/2/155/s1, Supplementary file 1: Search terms and flowcharts.
