# Supplementary material for: Assessing Scientific Soundness and Translational Value of Animal Studies on DPP4 Inhibitors for Treating Type 2 Diabetes Mellitus
Source: Biology (Basel). 2021 Feb 16;10(2):155. doi: 10.3390/biology10020155 (PMC7920304; doi:10.3390/biology10020155)

Supplementary file 1: Search terms and flowcharts

Assessing scientific soundness and translational value of animal studies on DPP4 inhibitors for treating type 2 diabetes mellitus

Nuno Henrique Franco [1, 2], Sonia Miranda Batista [1,2], Nóra Kovács [3], Attila Nagy [4] Bùi Quốc Thiện [5], Flávio Reis[6-8] and Orsolya Varga[3,9]*

[1] Laboratory Animal Science group, IBMC – Instituto de Biologia Molecular e Celular, Universidade do Porto, Rua Alfredo Allen 208, 4200-135 Porto, Portugal; nfranco@ibmc.up.pt; soniabatistamiranda@outlook.com>

[2] Instituto de Investigação e Inovação da Universidade do Porto, Rua Alfredo Allen 208, 4200-135 Porto, Portugal; nfranco@ibmc.up.pt; soniabatistamiranda@outlook.com

[3] Department of Public Health and Epidemiology, Faculty of Medicine, University of Debrecen, Kassai út 26, 4028 Debrecen, Hungary; kovacs.nora@med.unideb.hu; varga.orsolya@med.unideb.hu

[4] Faculty of Public Health, University of Debrecen, Kassai út 26, 4028 Debrecen, Hungary; nagy.attila@sph.unideb.hu

[5] Faculty of Medicine, University of Debrecen, Egyetem square 1, 4032 Debrecen, Hungary 4032; buiquocthien1996@gmail.com

[6] Institute of Pharmacology & Experimental Therapeutics, & Coimbra Institute for Clinical and Biomedical Research (iCBR), Faculty of Medicine, University of Coimbra, 3000-548 Coimbra, Portugal.; freis@fmed.uc.pt

[7] Center for Innovative Biomedicine and Biotechnology (CIBB), University of Coimbra, 3004-504 Coimbra, Portugal.; freis@fmed.uc.pt

[8] Clinical Academic Center of Coimbra (CACC), 3004-504 Coimbra, Portugal.; freis@fmed.uc.pt

[9] Office for Research Groups Attached to Universities and Other Institutions, Hungarian Academy of Sciences, 1051 Budapest, Hungary.; varga.orsolya@med.unideb.hu

A. Search terms

Human search terms

"Vildagliptin"[Mesh] AND (("placebos"[MeSH Terms] OR "placebos"[All Fields] OR "placebo"[All Fields]) AND controlled[All Fields]) AND (("glucose"[MeSH Terms] OR "glucose"[All Fields]) OR ("glycated hemoglobin a"[MeSH Terms] OR "glycated hemoglobin a"[All Fields] OR "hba1c"[All Fields])) AND Clinical Trial[ptyp] 62

'non insulin dependent diabetes mellitus'/exp AND 'vildagliptin'/exp AND ('glucose'/exp OR 'hemoglobin a1c'/exp) AND 'clinical trial'/exp AND placebo AND controlled AND ('human'/de OR 'randomized controlled trial'/de) AND 'article'/it AND 'placebo'/de

placebo | Completed Studies | Type 2 Diabetes | Vildagliptin | glucose OR HbA1c

///

(("placebos"[MeSH Terms] OR "placebos"[All Fields] OR "placebo"[All Fields]) AND controlled[All Fields]) AND (("Sitagliptin Phosphate"[Mesh] AND "Diabetes Mellitus, Type 2"[Mesh]) AND ("Glucose"[Mesh] OR "Glycated Hemoglobin A"[Mesh])) AND Clinical Trial[ptyp] 86

'non insulin dependent diabetes mellitus'/exp AND 'sitagliptin'/exp AND ('glucose'/exp OR 'hemoglobin a1c'/exp) AND 'clinical trial'/exp AND placebo AND controlled AND ('human'/de OR 'randomized controlled trial'/de) AND 'article'/it AND 'placebo'/de

placebo | Completed Studies | Type 2 Diabetes | Sitagliptin | glucose OR HbA1c

///

(("placebos"[MeSH Terms] OR "placebos"[All Fields] OR "placebo"[All Fields]) AND controlled[All Fields]) AND ((alogliptin AND "Diabetes Mellitus, Type 2"[Mesh]) AND ("Glucose"[Mesh] OR "Glycated Hemoglobin A"[Mesh]))

'non insulin dependent diabetes mellitus'/exp AND 'alogliptin'/exp AND ('glucose'/exp OR 'hemoglobin a1c'/exp) AND 'clinical trial'/exp AND placebo AND controlled AND ('human'/de OR 'randomized controlled trial'/de) AND 'article'/it AND 'placebo'/de

placebo | Completed Studies | Type 2 Diabetes | Alogliptin | glucose OR HbA1c

//

(("placebos"[MeSH Terms] OR "placebos"[All Fields] OR "placebo"[All Fields]) AND controlled[All Fields]) AND ("linagliptin"[MeSH Terms] AND "Diabetes Mellitus, Type 2"[Mesh]) AND ("Glucose"[Mesh] OR "Glycated Hemoglobin A"[Mesh]) AND Clinical Trial[ptyp]

'non insulin dependent diabetes mellitus'/exp AND 'linagliptin'/exp AND ('glucose'/exp OR 'hemoglobin a1c'/exp) AND 'clinical trial'/exp AND placebo AND controlled AND ('human'/de OR 'randomized controlled trial'/de) AND 'article'/it AND 'placebo'/de

placebo | Completed Studies | Type 2 Diabetes | Linagliptin | glucose OR HbA1c

///

(("placebos"[MeSH Terms] OR "placebos"[All Fields] OR "placebo"[All Fields]) AND controlled[All Fields]) AND ((("saxagliptin"[Supplementary Concept] OR "saxagliptin"[All Fields]) AND "Diabetes Mellitus, Type 2"[Mesh]) AND ("Glucose"[Mesh] OR "Glycated Hemoglobin A"[Mesh])) AND Clinical Trial[ptyp]

'non insulin dependent diabetes mellitus'/exp AND 'saxagliptin'/exp AND ('glucose'/exp OR 'hemoglobin a1c'/exp) AND 'clinical trial'/exp AND placebo AND controlled AND ('human'/de OR 'randomized controlled trial'/de) AND 'article'/it AND 'placebo'/de

placebo | Completed Studies | Type 2 Diabetes | Saxagliptin | glucose OR HbA1c

Animal search terms

Embase: sitagliptin AND animal experiment AND glucose OR hemoglobin a1c

PubMed: sitagliptin AND ("Hemoglobin A, Glycosylated"[Mesh] OR "Blood Glucose"[Mesh])) AND "animals"[MeSH Terms:noexp] AND (Animals[Mesh:noexp])

Web of Science: Topic=(sitagliptin) AND Topic=(blood glucose OR hemoglobin A) AND Topic=(animal). Search filter for animals suggested by Carlijn R Hooijmans.

///

Embase: saxagliptin AND animal experiment AND glucose OR hemoglobin a1c
PubMed: saxagliptin AND ("Hemoglobin A, Glycosylated"[Mesh] OR "Blood Glucose"[Mesh])) AND "animals"[MeSH Terms:noexp] AND (Animals[Mesh:noexp])
Web of Science: Topic=(saxagliptin) AND Topic=(blood glucose OR hemoglobin A) AND Topic=(animal). Search filter for animals suggested by Carlijn R Hooijmans.
//
Embase: linagliptin AND animal experiment AND glucose OR hemoglobin a1c
PubMed: linagliptin AND ("Hemoglobin A, Glycosylated"[Mesh] OR "Blood Glucose"[Mesh])) AND "animals"[MeSH Terms:noexp] AND (Animals[Mesh:noexp])
Web of Science: Topic=(linagliptin) AND Topic=(blood glucose OR hemoglobin A) AND Topic=(animal). Search filter for animals suggested by Carlijn R Hooijmans.
//
Embase: vildagliptin AND animal experiment AND glucose OR hemoglobin a1c
PubMed: vildagliptin AND ("Hemoglobin A, Glycosylated"[Mesh] OR "Blood Glucose"[Mesh])) AND "animals"[MeSH Terms:noexp] AND (Animals[Mesh:noexp])
Web of Science: Topic=(vilagliptin) AND Topic=(blood glucose OR hemoglobin A) AND Topic=(animal). Search filter for animals suggested by Carlijn R Hooijmans.
//
Embase: alogliptin AND animal experiment AND glucose OR hemoglobin a1c
PubMed: alogliptin AND ("Hemoglobin A, Glycosylated"[Mesh] OR "Blood Glucose"[Mesh])) AND "animals"[MeSH Terms:noexp] AND (Animals[Mesh:noexp])
Web of Science: Topic=(alogliptin) AND Topic=(blood glucose OR hemoglobin A) AND Topic=(animal). Search filter for animals suggested by Carlijn R Hooijmans

B. Flow charts

Alogliptin

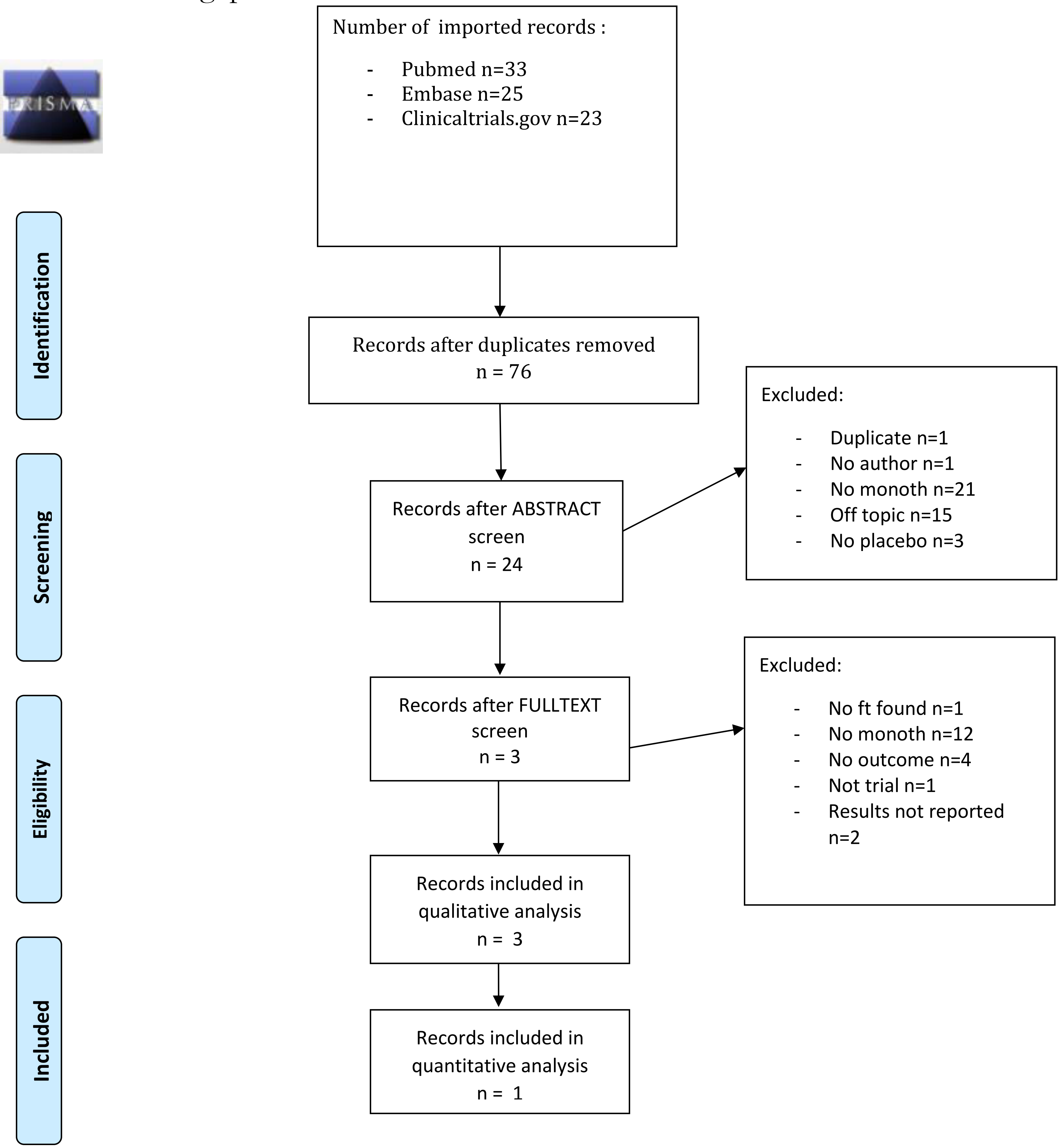

Linagliptin

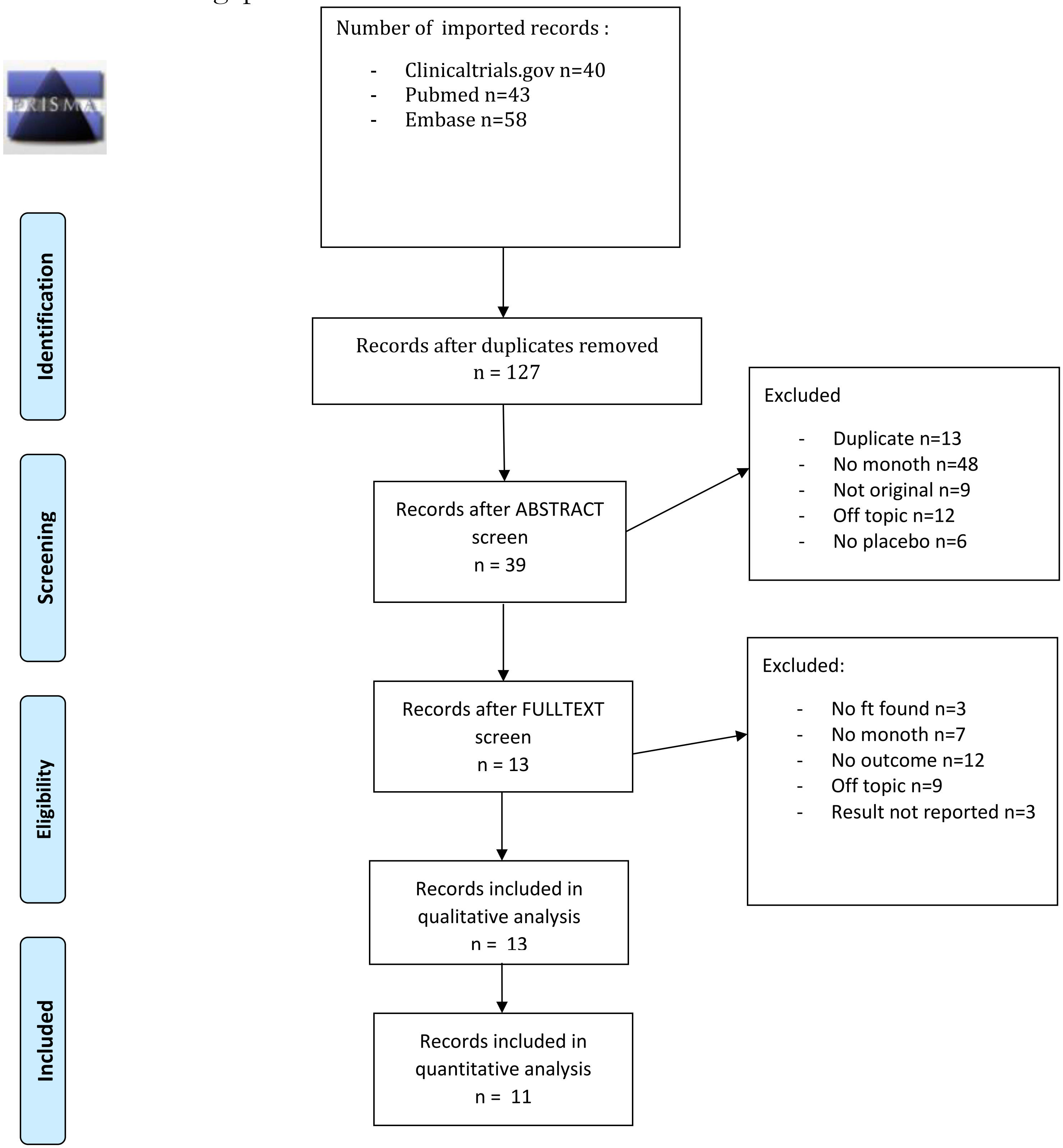

Saxagliptin

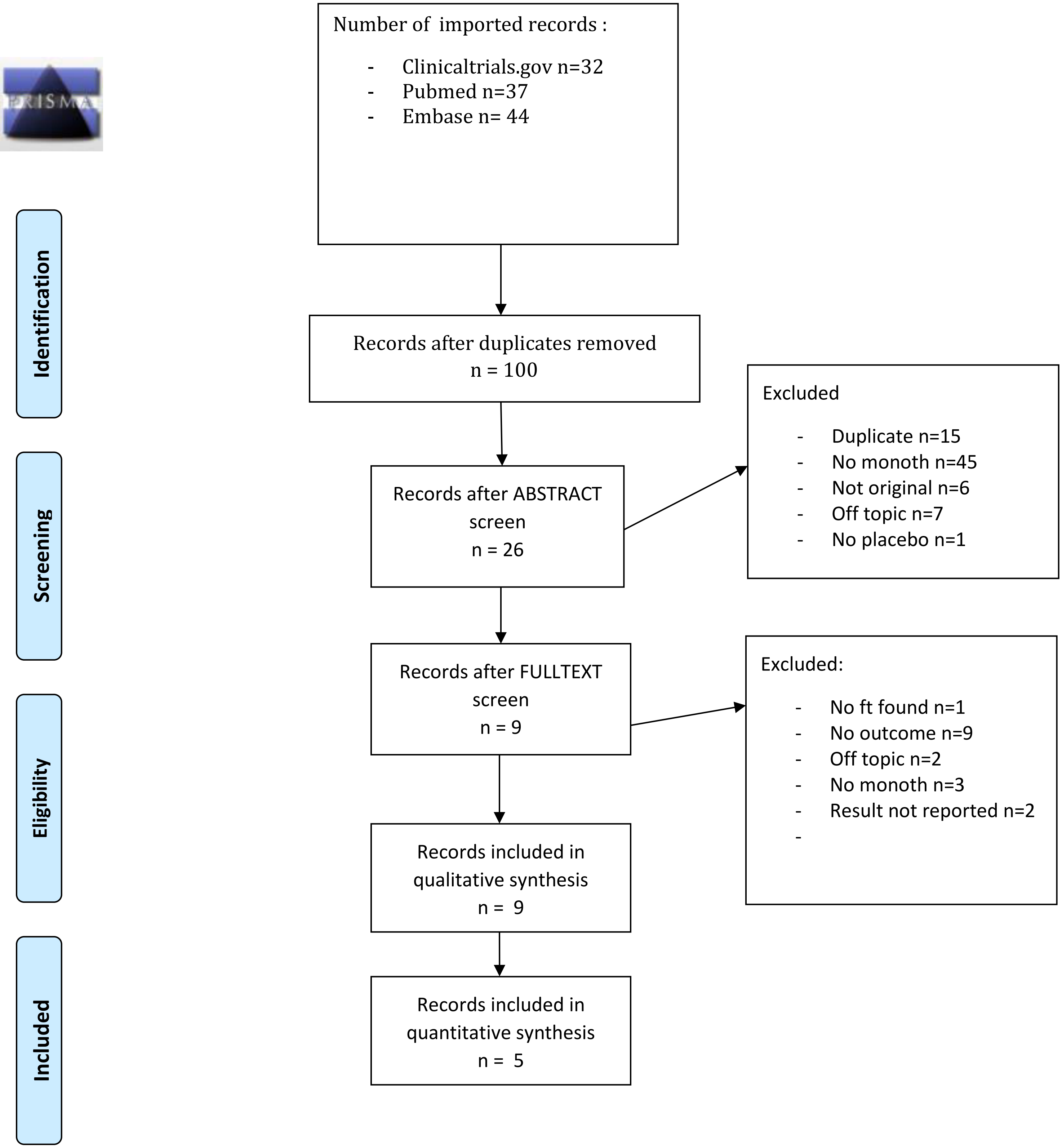

Sitagliptin

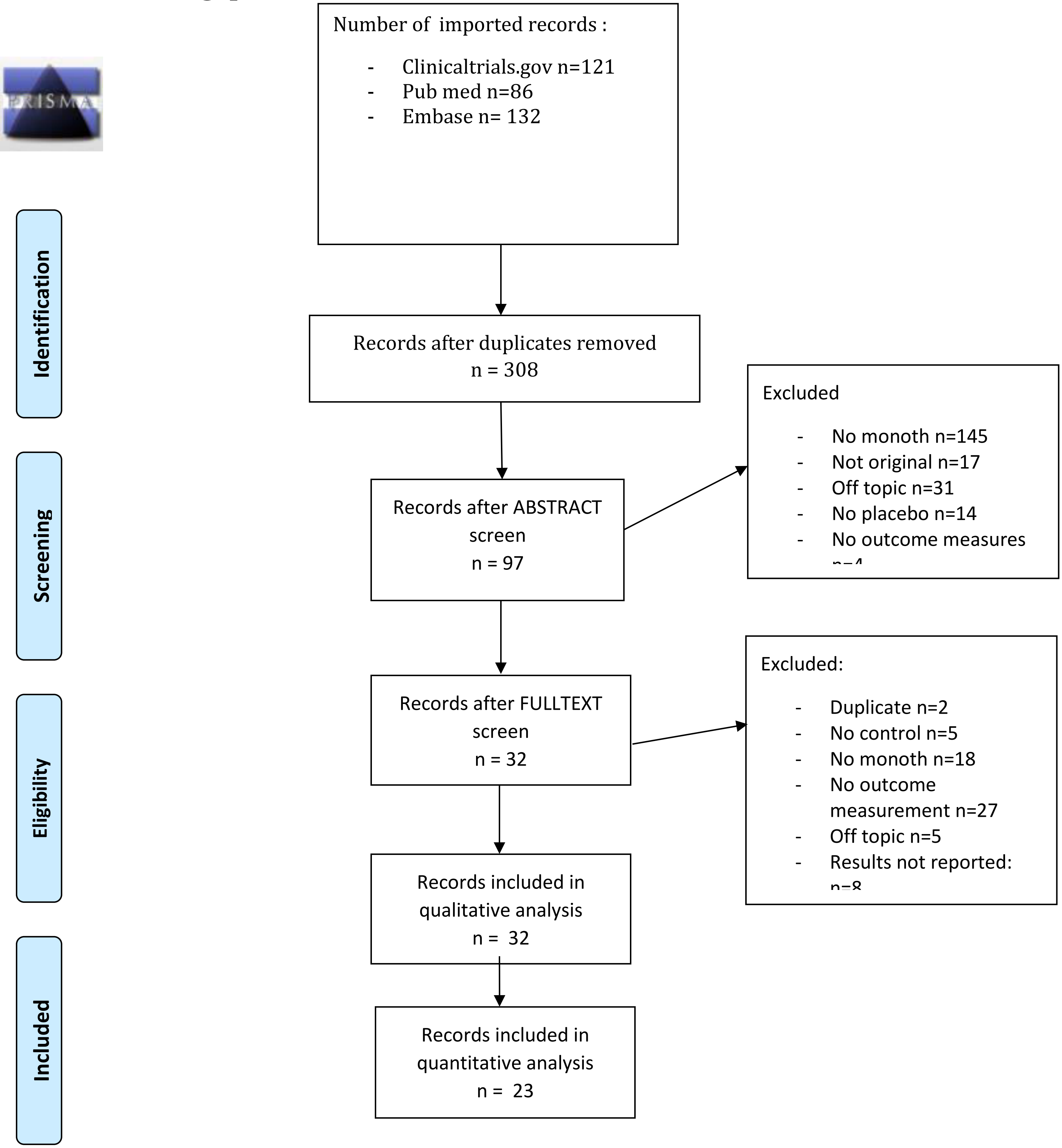

Vildagliptin

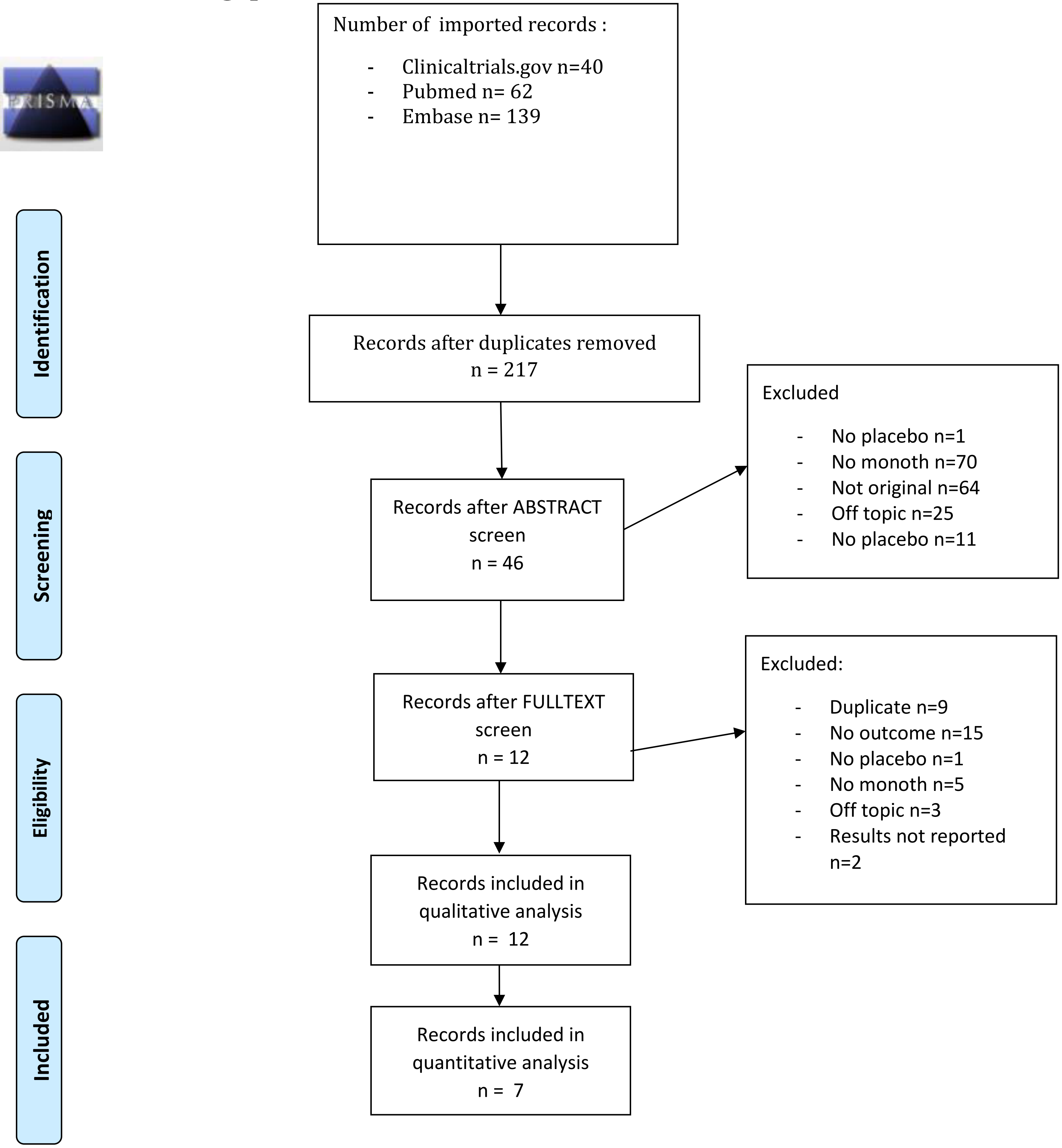

ALOGLIPTIN animal studies

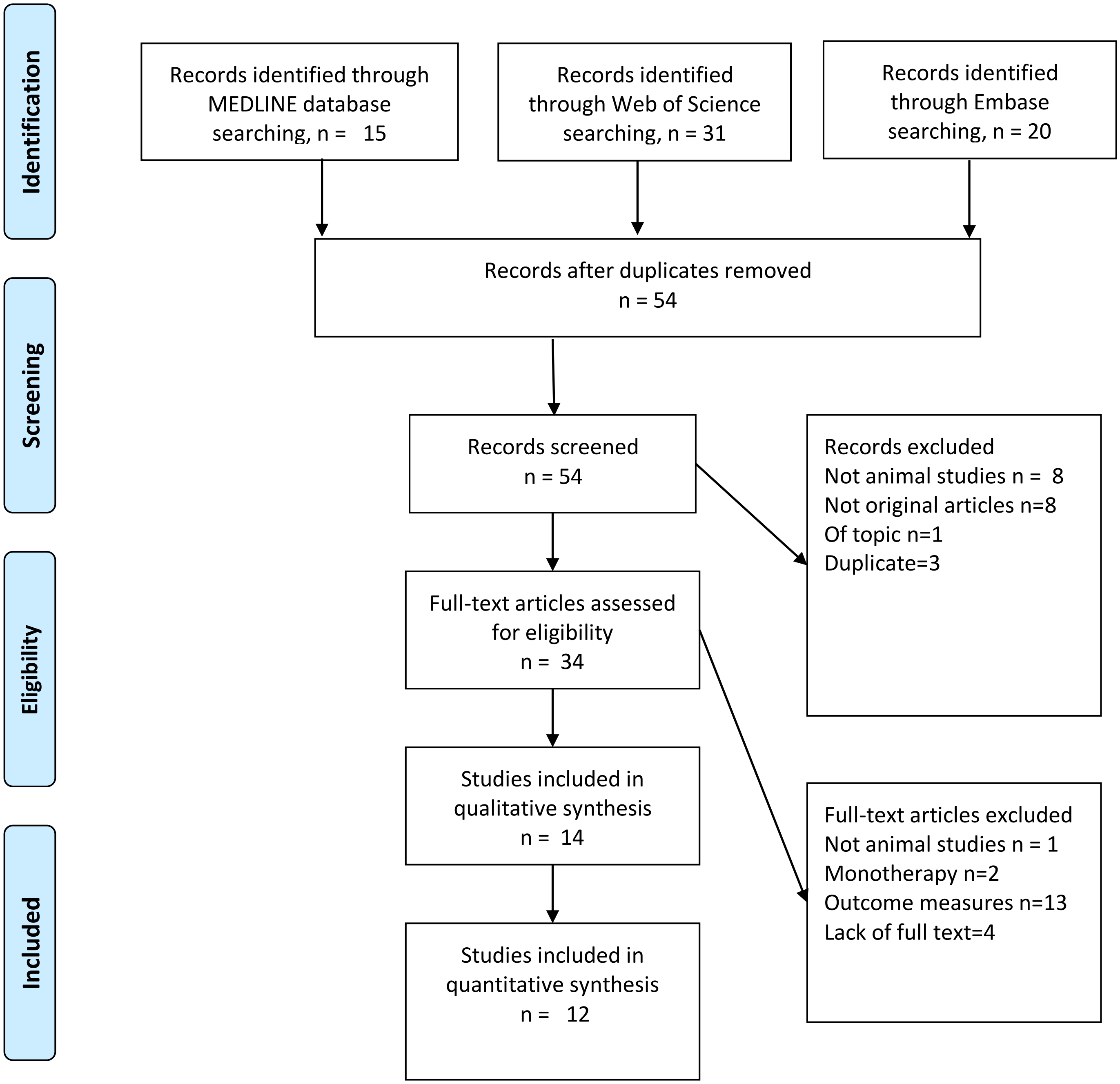

LINAGLIPTIN animal studies

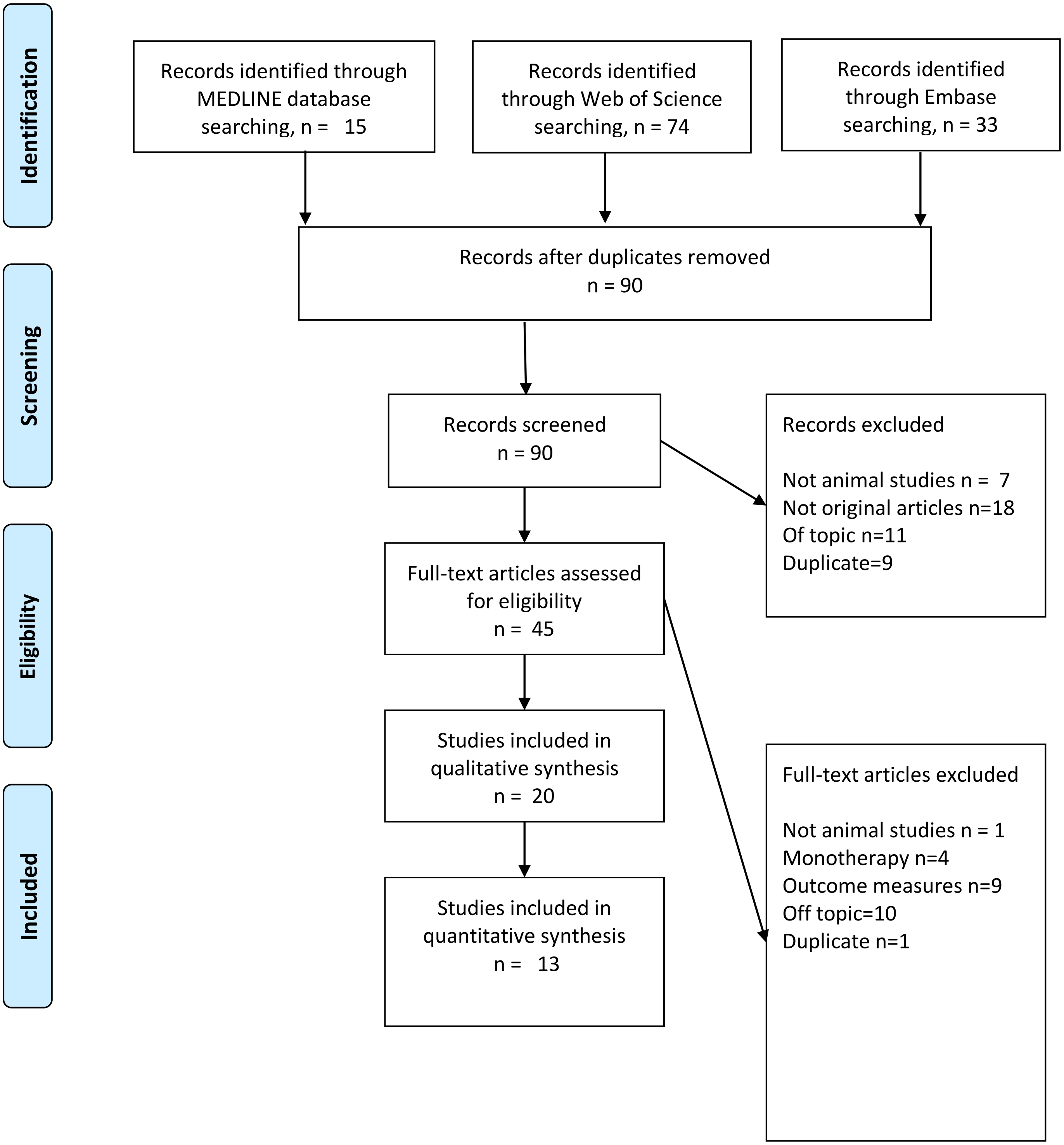

SAXAGLIPTIN animal studies

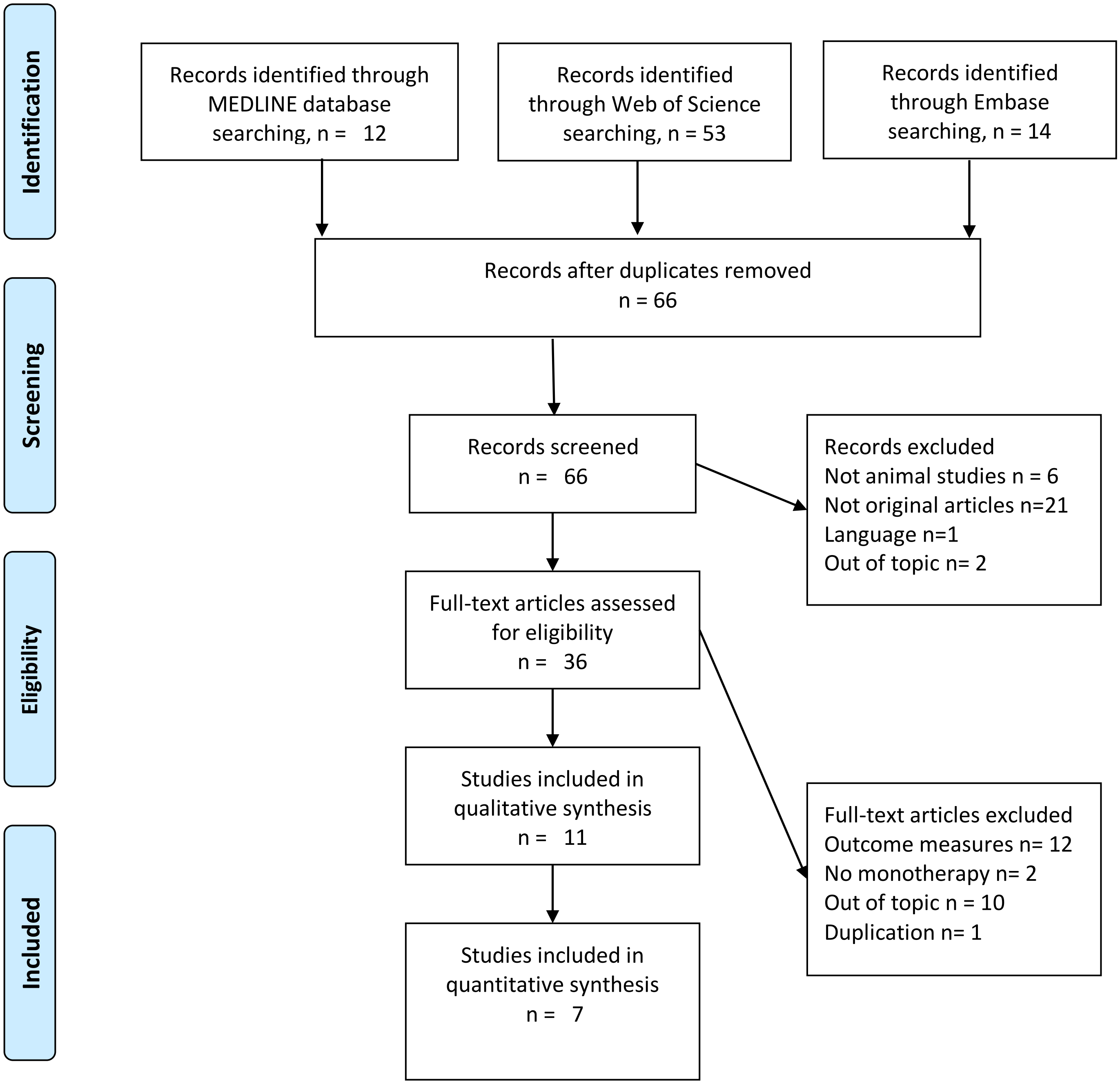

SITAGLIPTIN animal studies

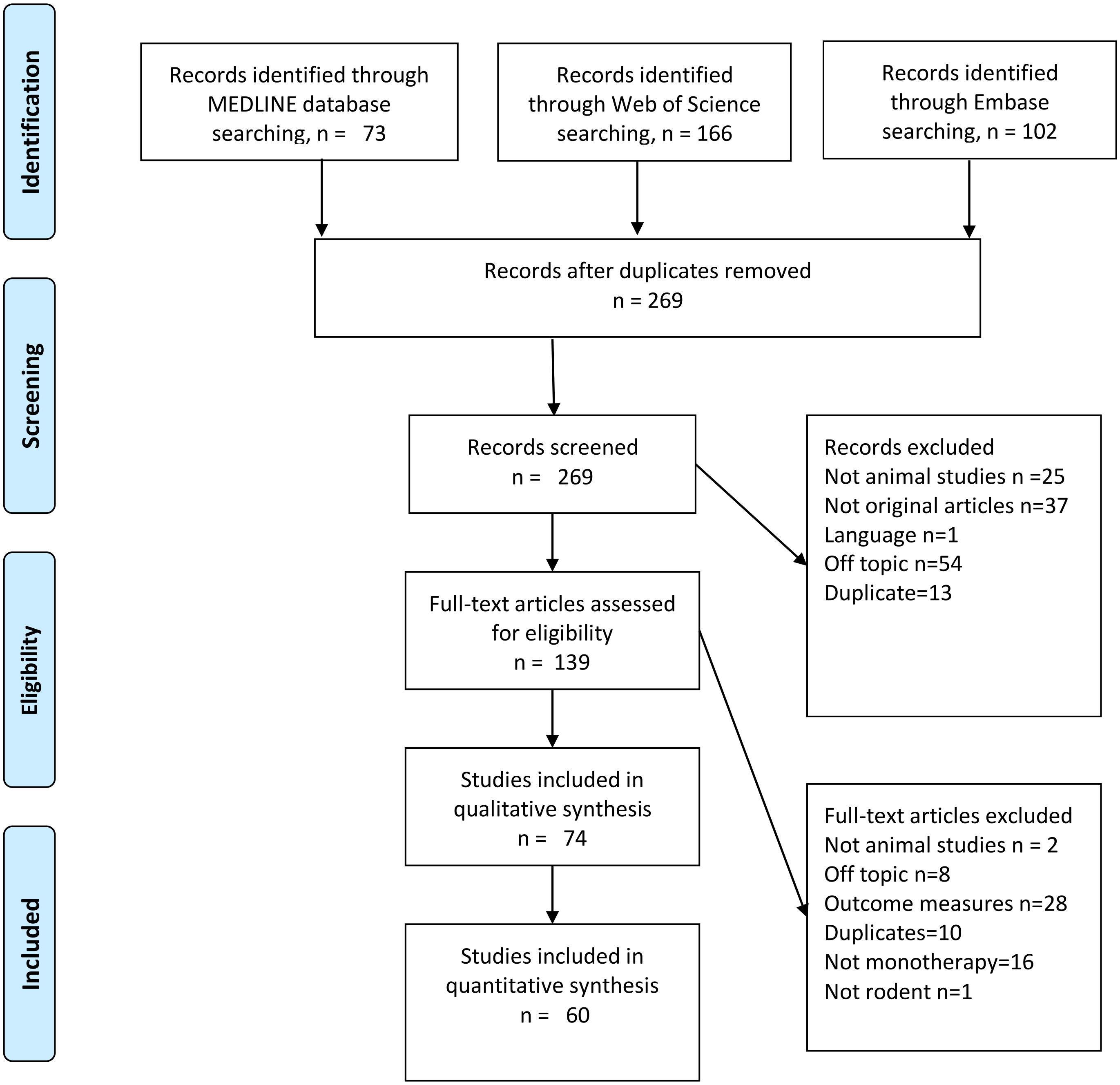

VILDAGLIPTIN animal studies

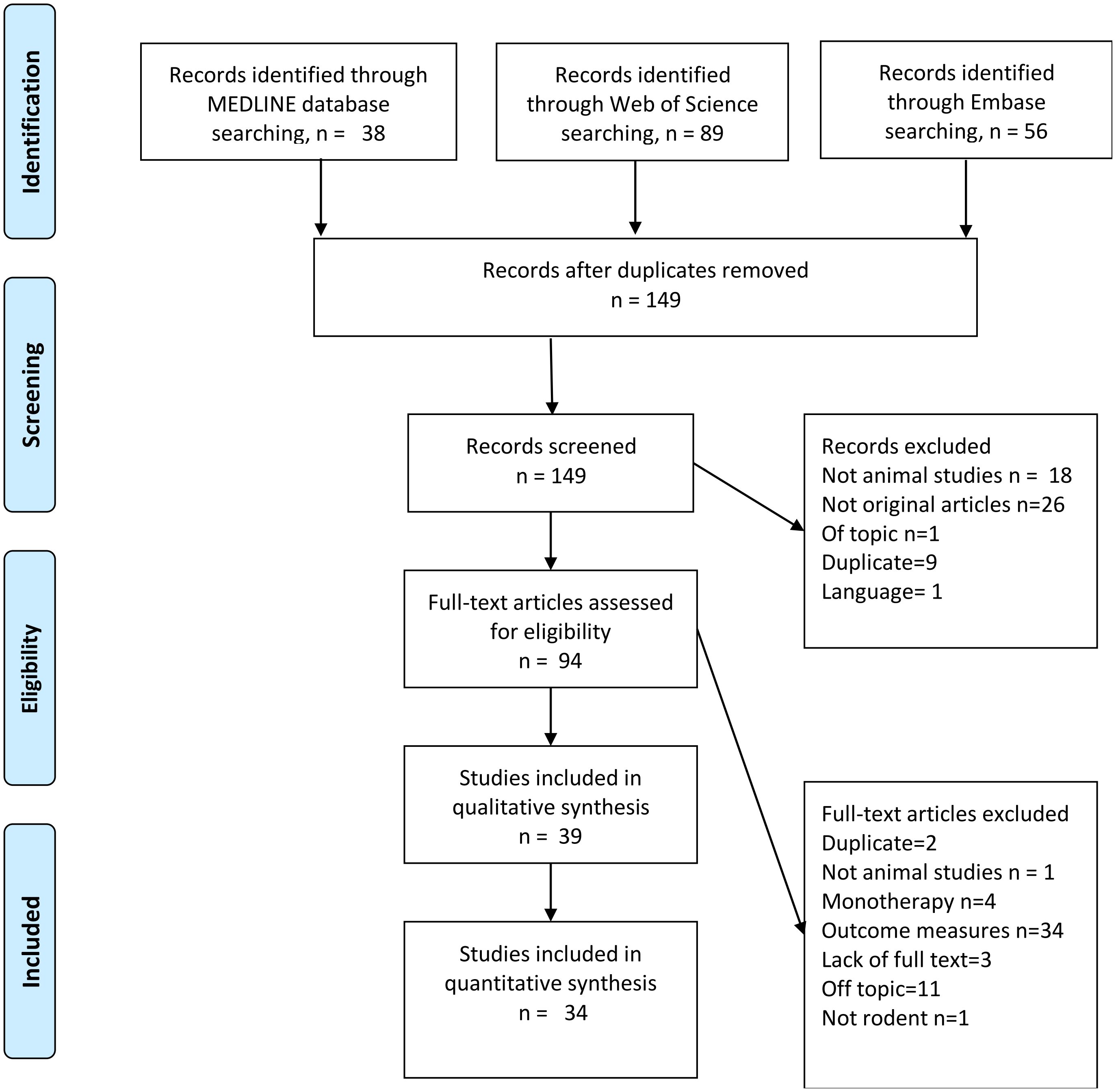

Supplement: Supplementary file 1 [file biology-10-00155-s001.pdf]
